# Combined effect of new complete dentures and simple dietary advice on nutritional status in edentulous patients: study protocol for a randomized controlled trial

**DOI:** 10.1186/s13063-016-1664-y

**Published:** 2016-11-09

**Authors:** Yuriko Komagamine, Manabu Kanazawa, Maiko Iwaki, Ayami Jo, Hiroyuki Suzuki, Noriko Amagai, Shunsuke Minakuchi

**Affiliations:** 1 Gerodontology and Oral Rehabilitation, Graduate School of Medical and Dental Sciences, Tokyo Medical and Dental University, Yushima, Bunkyo-ku, Tokyo, 113-8549 Japan; 2 Oral Diagnosis and General Dentistry, University Hospital of Dentistry, Tokyo Medical and Dental University, Tokyo, Japan

**Keywords:** Complete dentures, Edentulous, Nutrition, Dietary counseling, Dietary advice

## Abstract

**Background:**

Individuals who are edentulous have a lower intake of fruit, vegetables, fiber, and protein compared with their dentate counterparts because tooth loss is accompanied by a decrease in ability to chew. Whether or not a combination of prosthetic rehabilitation and simple dietary advice produces improvement in dietary intake among edentulous persons is unclear. We aim to investigate the effect of a simultaneous combination of simple dietary advice delivered by dentists and provision of new complete dentures on dietary intake in edentulous individuals who request new dentures.

**Methods/design:**

Through a double-blinded, parallel, randomized controlled trial in which 70 edentate persons who request new complete dentures will be enrolled, eligible study participants will be randomly allocated to either a dietary intervention group receiving dietary advice or to a control group receiving only advice on the care and maintenance of dentures. Outcome measures include daily intake of nutrients and food items, assessed using a brief self-administered diet history questionnaire; antioxidant capacity, determined using blood and urine samples; nutritional status, assessed with the Mini-Nutritional Assessment-Short Form; oral health-related quality of life, assessed with the Japanese version of the Oral Health Impact Profile-EDENT and the Geriatric Oral Health Assessment Index; subjective chewing ability; masticatory performance, assessed using a color-changeable chewing gum and a gummy jelly; patient self-assessment of dentures; mild cognitive impairment, assessed with the Japanese version of the Montreal Cognitive Assessment; and functional capacity, assessed with the Japan Science and Technology Agency Index of Competence. Outcome measures, except for antioxidant capacity, are to be implemented at three time points: at baseline and at 3 and 6 months following intervention. Antioxidant capacity data are to be collected twice: at baseline and at 3 months following intervention. Differences between the groups at 3 and 6 months and within-group changes are to be compared using the paired *t* test.

**Discussion:**

Simple dietary advice that can be implemented by a dentist would be more practical in clinical practice than tailored dietary counseling. The results of this study will provide beneficial information on dietary intake changes for both edentulous individuals requesting new complete dentures and dentists.

**Trial registration:**

University Hospital Medical Information Network Center Unique Trial Number: UMIN000017879. Registered on 12 June 2015.

## Background

Edentulous individuals consume significantly lower amounts of protein and multiple other nutrients, including fiber, calcium, and some vitamins, because they avoid a number of food types, particularly fresh fruit and raw vegetables and other hard and tough foods, when compared with dentate individuals [1–4]. Several lines of evidence show a relationship between increased intake of some macronutrients and micronutrients and a reduced risk of certain illnesses. Most notably, there is strong evidence that a diet high in fruit and vegetables protects against obesity, diabetes, cardiovascular disease, and some cancers [1, 2]. Poorer food intake by edentulous individuals is therefore likely to lead to ill health. Moreover, inadequate nutrient intake because of tooth loss is associated with weight loss, which is a factor in frailty [5], so edentulous individuals are also at risk of becoming frail.

Previous research has shown that, in many cases, prosthodontic rehabilitation alone, such as complete dentures [1, 6–8], implant overdentures [2, 4, 9–11], and other prosthetic treatments [12–14], for older patients in the absence of tailored dietary counseling is inadequate to attain a significant improvement in nutritional status. Most researchers suggest that dietary counseling is necessary. There has been little research investigating the impact of providing dietary counseling to edentulous individuals wearing complete dentures [1, 3, 15–17]. In a randomized controlled study [16] aimed at edentulous individuals without problems with their current complete dentures or mandibular implant overdentures, tailored dietary counseling benefited both complete dentures wearers and mandibular implant overdenture wearers. Mandibular implant overdenture wearers showed moderately greater dietary improvements when compared with patients wearing conventional dentures. In another randomized controlled study [1] aimed at edentulous individuals who request new dentures, simultaneous tailored dietary counseling delivered by a nutritionist on two occasions and provision of new complete dentures in combination improved fruit and vegetable intake. On the other hand, a combination of use of a denture adhesive and simple dietary advice in the form of government-issued dietary pamphlets issued by dentists produced significant improvement in dietary intake among edentulous individuals wearing current complete dentures for more than one year; there was especially an improvement in the number of portions of fruit and vegetables [3].

There has been no research investigating whether simple dietary advice in pamphlet form combined with provision of new complete dentures improves dietary intake among edentulous individuals who request new dentures. Therefore, we plan to test the null hypothesis that there would be no difference in dietary intake between edentulous individuals provided with simple dietary advice and new complete dentures and edentulous individuals provided with new dentures alone.

## Methods/design

This double-blind, parallel, randomized controlled trial is investigating the effect of a combination of simple dietary advice and provision of new complete dentures on dietary intake in elderly edentulous individuals. The study follows the 2010 Consolidated Standards of Reporting Trials (CONSORT) statement [18]. The study protocol and informed consent documents have been approved by the institutional ethics committee at Tokyo Medical and Dental University (TMDU, registry number 1144). The trial is registered with the University Hospital Medical Information Network (UMIN) Center (UMIN-CTR Clinical Trial, Unique Trial Number: UMIN000017879).

All participants are required to give written informed consent. The study participants are to be randomly allocated either to the dietary intervention group or to the control group. All participants receive complete denture treatments.

### Participants

We are recruiting trial participants as follows:

- Respondents to flyers in the reception room of the prosthodontics clinic at the University Hospital of Dentistry, TMDU
- Respondents to advertisements in geriatric magazines
- Respondents to an announcement on a website
- Patients who have received treatment at the undergraduate student treatment clinics at the University Hospital of Dentistry, TMDU
- New patients at the University Hospital of Dentistry, TMDU

### Eligibility

Participants will undergo screening to check that they meet the inclusion criteria for the study listed below. Those deemed to be eligible will be asked to give written informed consent for participation in the study before entry into the trial. They will be informed that it is possible to stop participating in the research at any time, and that there will be absolutely no penalty for requesting to stop. The study will be explained to eligible participants by the two outcome assessors (HS and NA), who will also obtain consent directly from the participants. Next, age, gender, family income, education, personal living circumstances, duration of edentulousness, number of previous complete dentures, and degree of ridge resorption are recorded. Gerotranscendence status is then assessed with the Japanese Gerotranscendence Scale Revised (JGS-R) [19, 20], and denture quality by functional assessment of dentures [21, 22] is recorded. In addition, outcome measures are to be assessed at baseline.

#### Inclusion criteria

Each participant included in this study must:

- Have made a request for new both upper and lower dentures for a dental problem
- Be able to understand the explanations and instructions given by clinicians or outcome assessors
- Be able to understand and respond to the questionnaires used in the study
- Have the means to control any recommended dietary change

#### Exclusion criteria

A potential participant will be excluded if he/she has:

- An infectious disease
- An orofacial motor disorder
- A psychiatric disorder
- A diagnosis of dementia
- Any dietary restrictions
- No means to implement dietary change, e.g., a person living in an institution

### Interventions

All patients receive complete denture treatments. The technique used to create a complete denture involves primary and secondary impressions, recording of jaw relationships using occlusal rims, one or two trial insertions, and delivery of the new complete dentures. After delivery, adjustments to the dentures are made until both participant and clinician deem that no further adjustment is necessary. The new complete dentures are made by three dentists with 10–14 years of clinical experience (YK, MK, and MI). They will give complete denture treatments, but will neither deliver dietary advice or advice on care and maintenance of dentures nor assess outcomes. Participants will be randomly assigned to the three dentists.

Participants in the dietary intervention group receive 20 minutes of coordinated dietary advice in the form of an explanation of a pamphlet prepared with reference to the geriatric version of the Japanese Food Guide Spinning Top published by the Japanese Ministry of Agriculture, Forestry and Fisheries. The pamphlet is then given to the participant. If a participant does not prepare his or her own meals, the person who does the cooking receives the dietary advice as well.

Participants in the control group receive 20 minutes of coordinated advice for care and maintenance of dentures in the form of an explanation of a pamphlet prepared with reference to the guidelines published by the American Collage of Prosthodontics [23], after which the pamphlet is provided to the participant. The advice on diet and care and maintenance of dentures is provided by the two outcome assessors (HS and NA). The two-time deliveries of advice in the dietary intervention and control groups are designed to fit with dental appointments for try-insertion and delivery of new dentures.

### Outcome measures

**Fig. 1:**
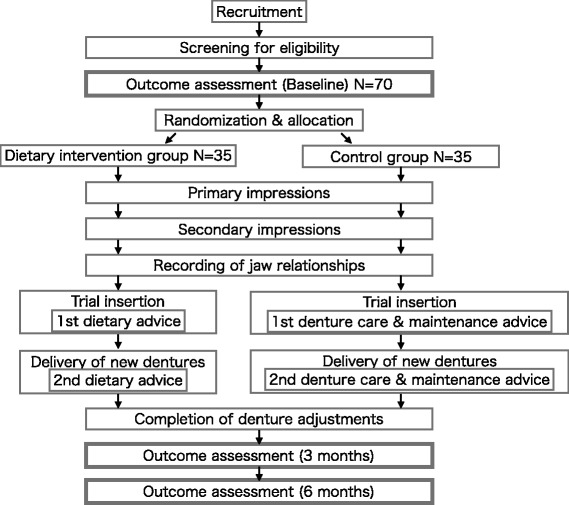
Chart of trial design

Outcome measures are assessed at baseline and at 3 and 6 months following the intervention, with the exception of antioxidant capacity (Fig. 1). Antioxidant capacity data are collected at baseline and at 3 months following the intervention. The outcome assessments are to be done by three dentists (AJ, HS, and NA). The participants will be paid as a research corporation expenditure when outcome measure at 6 months following the intervention is completed. The outcome assessors will revise all completed questionnaires before the participants leave to avoid missing data at the end of each assessment. The recorded data are quickly deidentified and digitized, and will be stored and managed for ten years according to the rules of the institutional ethics committee of Tokyo Medical and Dental University.

#### Primary outcome measures

The primary outcome measures are fruit, vegetable, and protein intake measured using a brief-type self-administered diet history questionnaire (BDHQ). The BDHQ enquires about the frequency of intake of 75 food items during the previous month. The daily intake of approximately 30 nutrients and about 50 food items is estimated. The validity and reliability of the BDHQ are established [24, 25].

#### Secondary outcome measures

##### Dietary intake

The daily intakes of nutrients and food items, except for the primary outcomes, are assessed using the BDHQ [24, 25].

##### Assessment of antioxidant capacity

Antioxidant capacity will be assessed at baseline and at 3 months following intervention. The antioxidant potential is determined using a blood sample, and 8-hydroxy-2′-deoxyguanosine, a marker of oxidative stress, is measured in a urine sample. These markers are sensitive to changes in dietary intake of fresh fruit and vegetables. A 5-mL blood sample from each participant is collected into a K2EDTA vacutainer, from which plasma is separated and stored at −73 °C prior to analysis. All participants are required to have fasted for more than 8 hours prior to sampling, and all blood samples are taken at noon. Participants are also required to provide a 3-mL sample of morning urine.

##### Nutritional status

Nutritional status is assessed with the Mini-Nutritional Assessment-Short Form (MNA-SF) [26]. The MNA-SF has the option of using calf circumference when body mass index is not available. A score between 8 and 11 indicates a risk of deficient nutrition, and scores lower than 7 indicate malnutrition.

##### Anthropometric measurements

Weight and body composition are assessed by bioelectrical impedance (Tanita Inner Scan BC-522; Tanita, Tokyo, Japan). Body composition is estimated by determining body fat percentage.

##### Oral health-related quality of life

Oral health-related quality of life is assessed using the Japanese versions of the Oral Health Impact Profile-EDENT [27] and the Geriatric Oral Health Assessment Index [28] questionnaires. The Japanese version of the Oral Health Impact Profile-EDENT contains 19 questions and seven conceptual subscales: functional limitation, pain, psychological discomfort, physical disability, psychological disability, social disability, and handicap. Higher scores indicate lower oral health-related quality of life. The Japanese version of the Geriatric Oral Health Assessment Index contains 12 questions and three conceptual fields: functional field, psychosocial field, and pain and discomfort field. Higher scores indicate better oral health-related quality of life.

##### Perceived chewing ability

Twenty food items are to be assessed for the participants’ perceived ability to bite and chew food using a four-point rating scale [29]. The ratio of number of food items that a participant answers “can chew well,” which represents the highest rating for the 20 food items, is calculated to give a score for self-perceived chewing ability.

##### Masticatory performance

Mixing ability is evaluated using a color-changeable chewing gum (Xylitol Masticatory Performance Evaluating Gum; Lotte Co., Ltd., Tokyo, Japan) [30]. The participants are instructed to chew the gum depending on their preference, using 100 chewing cycles, at a rate of once per second. Mixing ability is measured by a colorimeter. Shearing ability is evaluated using a test gummy jelly (Test Gummy Jelly for Evaluating Masticatory Performance; UHA Mikakuto Co., Ltd., Osaka, Japan) [31]. The participants are instructed to chew the test gummy jelly using 30 chewing cycles, depending on their preference. Shearing ability is measured by a visual scoring method [32].

##### Patient self-assessment of dentures

Patients make a self-assessment of their dentures using the Patient’s Denture Assessment (PDA) [33]. The PDA comprises 22 items covering six subscales classified as function, lower denture, upper denture, expectation, aesthetics and speech, and importance. Each item in the questionnaire is rated using a 100-mm visual analog scale.

##### Assessment of mild cognitive impairment

Mild cognitive impairment is to be assessed with the Japanese version of the Montreal Cognitive Assessment (MoCA-J) [34]. The MoCA-J comprises 16 items covering eight domains: visuospatial/executive, naming, memory, attention, language, abstraction, delayed recall, and orientation. Low educational attainment is corrected for by adding 1 point to the participant’s final score for ≤12 years of formal education. The highest possible score is 30, with a score of 26 or above indicating normal cognitive function.

##### Functional capacity

Maintenance of competence is evaluated with the Japan Science and Technology Agency Index of Competence (JST-Index of Competence), developed from the Tokyo Metropolitan Institute of Gerodontology Index of Competence [35–37]. The JST-Index of Competence comprises 16 items and four categories classified as utilization of new equipment, information collection, management of daily life, and social engagement. Higher scores indicate better functional status.

### Criteria for withdrawal

Patients will be withdrawn from the study in the event of any relevant deterioration in health likely to affect participation, if they withdraw their consent, or if they die.

### Sample size estimation

The sample size was determined based on the two primary outcomes: changes in the weight of fruit and vegetable intakes and in the weight of protein intake. In case of change in the weight of fruit and vegetable intakes, 29 individuals per group would provide 80 % power to detect a difference of 125 g per day in intake, with a two-sided test at a 0.05 level of significance for the primary outcome. In case of change in the weight of protein intake, 30 individuals per group would provide 80 % power to detect a difference of 15 g per day in protein intake, with a two-sided test at a 0.05 level of significance for the primary outcome. We also anticipated a 15 % rate of loss to follow-up. Accordingly, we adjusted our target baseline sample size to 35 per group.

### Randomization and allocation

Eligible study participants are to be randomly assigned to the dietary intervention group or the control group in an alternate fashion. The randomization sequence has been established with block sizes of 10 and an allocation ratio of 1:1 before recruitment commences and is to be concealed in opaque consecutively numbered envelopes. After the decision on participants’ enrollment, the envelopes will be opened. Patient enrollment and randomization will be undertaken by two outcome assessors (HS and NA). Participants and clinicians will be blinded to the group allocation of each participant. The participants will be given repeated instructions not to reveal their group allocation to clinicians. Any questions from participants during the intervention period will be answered by the outcome assessors (AJ, HS, and NA).

### Statistical analysis

All analyses are to be based on the initial treatment assignment (intention to treat). Missing data will be replaced with multiple imputation to primary and secondary outcome variables. Differences between the groups at 3 and 6 months and within-group changes are to be compared using the paired *t* test. All statistical analyses will be performed using IBM Statistical Package for the Social Sciences (SPSS) Statistics version 17.0 software. A significance level of 0.05 is used for each test.

## Discussion

This randomized controlled trial is designed to demonstrate the effect of simultaneous simple dietary advice and provision of new complete dentures in combination on dietary intake in edentulous individuals who request new dentures. It is hypothesized that a combination of provision of new complete dentures and simple dietary advice would improve nutrient intake among edentulous individuals.

Whereas in previous clinical research, nutritional intervention consisted of one-to-one counseling sessions with a nutritionist [1], the nutritional intervention in the present study consists of dentists giving participants a pamphlet along with a standardized detailed explanation of its contents. In a cohort study designed to determine if a combination of denture fixative use and dietary simple advice in the form of printed government-issued pamphlets would improve participants’ diets, fruit and vegetable intake was reported to improve over a 30-day period [3]. The researchers expected that this intervention would have a long-term effect on the basis that once a dietary choice is made, it is likely to continue.

Both participants and clinicians are blinded in this study so that neither will be aware of the allocated intervention. Nevertheless, the two outcome assessors (HS and NA) of the three outcome assessors (AJ, HS, and NA) will implement the randomization and allocation. These two outcome assessors also deliver advice, although they will not give complete denture treatments. Because these two outcome assessors are also delivering advice, neither outcome assessors nor those who deliver advice are blinded, which may cause bias. However, the bias against delivering advice has a smaller influence on the results of the research than the bias against giving treatments. Moreover, the outcomes assessed in this research consist of patient-reported outcomes and objective outcomes. Thus, only a small number of outcome assessors’ subjective outcomes are included, which leads to less possibility of observation bias.

An exclusion criterion for fruit and vegetable intakes at baseline is not set. Thus, participants whose fruit and vegetable intakes at baseline are already high might be included. However, it is reported that the mean consumption of vegetables in Japanese edentulous patients is lower than the Japanese government recommended vegetable intake of 350 g/d [38].

The effect of simple dietary advice alone in the absence of provision of complete dentures will not be investigated in this study. The previous study, aimed at complete denture wearers or implant overdenture wearers who did not need new dentures, showed that provision of tailored dietary counseling alone improves dietary intake in wearers of both types of dentures. In contrast, individuals who request new complete dentures are to be recruited in the present study. It would be unethical to withhold replacement of new dentures until completion of the study to investigate the effect of simple dietary advice alone in the absence of complete denture treatment. Therefore, the effect of simple dietary advice alone in the absence of provision of complete dentures will not be investigated in this study.

There is no opportunity for patients assigned to the control group to receive dietary advice; however, on completion of the study, the control group will receive the same dietary advice as the intervention group.

Simple dietary advice that can be implemented by a dentist is more practical in daily clinical practice than tailored dietary counseling, which requires the additional services of a nutritionist and a further appointment. The findings of this study will provide both patients and dentists with beneficial information with regard to improving dietary intake in edentulous individuals who request new complete dentures.

## Trial status

This trial is currently in the participant recruitment phase.

## Abbreviations

BDHQ: Brief-type self-administered diet history questionnaire
CONSORT: Consolidated Standards of Reporting Trials
GOHAI: Geriatric Oral Health Assessment Index
JGS-R: Japanese Gerotranscendence Scale Revised
JST-Index: Japan Science and Technology Agency Index
MNA-SF: Mini-Nutritional Assessment-Short Form
MoCA-J: Japanese version of Montreal Cognitive Assessment
PDA: Patient’s Denture Assessment
TMDU: Tokyo Medical and Dental University
TMIG-Index: Tokyo Metropolitan Institute of Gerodontology Index of Competence
UMIN: University Hospital Medical Information Network
